# An online case-based teaching and assessment program on clinical history-taking skills and reasoning using simulated patients in response to the COVID-19 pandemic

**DOI:** 10.1186/s12909-022-03950-2

**Published:** 2023-01-04

**Authors:** Barbara Duffy, Roisin Tully, Alice V. Stanton

**Affiliations:** Schools of Medicine, Pharmacy and Biomolecular Sciences, Royal College of Surgeons, Dublin, Ireland

**Keywords:** COVID-19 pandemic, Online teaching, Online assessment, Case-based, Simulated patients, Technology

## Abstract

**Background:**

The COVID-19 pandemic has created unprecedented challenges for medical students and educators worldwide. Groups 1, 2 and 3 of year 3, semester 2 medical students at the Royal College of Surgeons in Ireland (*n* = 275) had only completed 2, 5 and 7 weeks, respectively, of their scheduled 10-week clinical medicine and surgery attachments, prior to the Irish shutdown of all in-person non-essential activities, including medical student education.

**Methods:**

We developed and delivered an online case-based program, focused on history-taking skills and clinical reasoning, using simulated patients and video technologies. 12 tutorials were delivered over 6 weeks to 35 subgroups of 8 students in line with program learning outcomes. Both simulated patients (*n* = 36), and tutors (*n* = 45, from retired clinical professors to newly graduated physicians), were rapidly upskilled in Blackboard Collaborate and Microsoft Teams, and also in the provision of constructive feedback. We evaluated this newly developed program by the following three criteria: student attendance, achieved grades, and student feedback.

**Results:**

Attendance at the 12 tutorials was higher amongst group 1 and 2 students (75 and 73%) by comparison with group 3 students (60%) (p = < 0.001). Of the 273 students that sat the Year 3 Semester 2 online long case assessment, 93% were successful. Despite group 1 students having the least prior clinical experience, results were similar to those of groups 2 and 3 (1st honors, 2nd honors, pass, and fail grades for group 1, 39%, 33%, 23% and 6%; group 2, 34%, 41%, 17% and 8%; group 3, 39%, 25%, 28% and 7%) (*p* = 0.48). An increased attendance rate at tutorials was associated with higher numbers of honors grades (p = < 0.001). Anonymous feedback from the students demonstrated considerable satisfaction with program: > 85% agreed that the online program was interactive and very educational.

**Conclusions:**

Use of online video technology, tutors of varied experience, and simulated patients were demonstrated to replicate patient encounters, and to facilitate the development of clinical skills remotely during the COVID-19 pandemic.

**Supplementary Information:**

The online version contains supplementary material available at 10.1186/s12909-022-03950-2.

## Background

The emergence of COVID-19 has created unprecedented challenges for medical students and educators internationally [1]. The delivery of clinical attachments, used to orientate students to the clinical setting, develop communication, history taking, and examination skills, has faced unique challenges [2]. The acquisition of these skills is important as it imparts on students not only clinical skills but fundamental elements of their professional selves [3]. Traditionally this teaching has happened at the bedside, on the ward and in the out-patient clinic with active teaching and role-modeling [4]. Confronted with the closure of universities, the redeployment of academic staff to the frontline and cessation of clinical attachments, medical schools worldwide have had to rapidly implement significant changes to the delivery of traditional curriculum [2, 5–7]. This challenge has seen innovative solutions especially with the use of the virtual environment [8]. Universities have responded to the limitations, due to the enforcement of restrictions during the COIVD-19 pandemic, with programs that incorporate elements of eLearning, online distance education, recorded videos, podcasts, flipped classrooms, synchronous and asynchronous learning and delivery of online examinations through platforms such as Zoom and MS Teams [1, 9]. A novel way students in Hong Kong developed and practiced clinical history taking skills was with a chatbot mobile app. Assessment of student performance in clinical history taking was comparable to those in a group taught using conventional methods [10]. Other universities that had some access to clinical encounters in the out-patient environment but no longer in the hospital setting supplemented skill acquisition with online video-based learning [11]. Despite many negative aspects, lessons learned from the COVID-19 pandemic may serve as a catalyst for change and reform of medical education going forward [12].

In response to Irish Governmental restrictions, the Royal College of Surgeons Ireland (RCSI) announced the closure of its Dublin campuses on March 12th 2020, suspending face-to-face teaching and clinical attachments. International students were advised to return to their home countries, while academic and administrative staff began devising plans to pivot to remote working and teaching across a range of programs. At this time, the year 3 semester 2 students in RCSI had only completed a fraction of the teaching program. Following the university closure, the faculty team rapidly developed an online case-based teaching program within 3 weeks. The aims of the newly developed online teaching program were to:

- Continue clinical teaching and learning opportunities remotely during the COVID-19 Pandemic;
- Address the Clinical Medicine and Surgery module learning objectives; and
- Facilitate completion of the year 3 semester 2 long case examination online and facilitate onward progression through the undergraduate medical program.

In this paper we share our experience of transitioning a clinical module to a digital platform in terms of program design, implementation and resources required. We discuss the evaluation of our program in terms of student attendance, assessment results and the results of anonymous student feedback. We also discuss future directions for online or blended clinical teaching in medical programs based on our findings.

## Methods

### Educational setting and the context of the learners

This study took place in the Royal College of Surgeons Ireland (RCSI), University of Medicine and Health Sciences in Dublin, in semester 2 of year 3 of a 5 year undergraduate bachelor degree. In years 1 and 2 and semester 1 of year 3, there had been a focus on covering the basic health sciences (anatomy, physiology, biochemistry, pharmacology, epidemiology, microbiology and pathology), supplemented with some case based teaching and clinical skills development. The year 3 semester 2 program marks students’ entry into full time clinical attachments for the first time. To align with this they are enrolled in two modules: the Clinical Medicine and Surgery (CMS) module, which involves 10 weeks of clinical attachment, and the Student Selected Project (SSP). The SSP is a six-week block in which students carry out a research project under an appropriate supervisor, and subsequently produce a report and presentation of their project. This was also extensively modified due to the COVID-19 restrictions but is not dealt with in this paper.

Table 1 details the standard components of the teaching program. The aim of the CMS module is to gain the appropriate knowledge, clinical skills and professionalism to allow the accurate diagnosis and optimal management of common medical and surgical conditions. Clinical teaching during the semester is balanced with a mixture of experiential learning on clinical attachments, bedside teaching of clinical skills, didactic sessions on practical skills, professionalism and communication, in addition to self-directed research. The musculoskeletal (MSK) multiple choice question (MCQ) exam is a knowledge based assessment of musculoskeletal theory covered within the 2 weeks of didactic teaching (Table 1). This was run online in response to COVID-19 restrictions but is not dealt with in this paper. The Objective Structured Clinical Examination (OSCE) is a clinical skills examination involving the examination of real patients. This component of the CMS module was deferred until face to face teaching recommenced in September 2020.

**Table 1:** The standard structure of the year 3 semester 2 program

			Group 1 (*n* = 106)	Group 2 (*n* = 86)	Group 3 (*n* = 83)
Clinical Medicine and Surgery Module (CMS) (12 weeks)	Didactic teaching (2 weeks)		✓	✓	✓
	Clinical attachments		2 of 10 weeks	5 of 10 weeks	7 of 10 weeks
	Examination	Long case	✕	✕	✕
		OSCE	✕	✕	✓
		MSK MCQ	✕	✓	✕
Student selected project (SSP)(6 weeks)			✓	✕	✕

The degree of completion of each component by each group at time of the COVID-19 restrictions is listed. ✓ = completed, ✕ = not completed

A total of 275 students were divided into three groups to facilitate rotating through clinical placements. At the time of the University closure in March 2020, each group of students had varying levels of clinical experience (2, 5 and 7 weeks for Groups 1, 2 and 3 respectively) (Table 1).

### Online program design

A six-week online program focusing on communication and history taking skills was developed and delivered via teaching sessions using Blackboard Collaborate (Blackboard Inc., Washington, DC, USA). The novel online learning program focused on five of the original eight CMS module learning outcomes that address history-taking skills (using the Calgary Cambridge model [13]), and clinical reasoning (Table 2).

**Table 2:** The original CMS module learning outcomes and the online program learning outcomes

CMS module learning outcomes	
Original CMS Learning Outcomes	Online program learning outcomes
Obtain a complete and accurate patient-centred history for common respiratory, cardiac, gastrointestinal, neurological, genitourinary, vascular, abdominal, and endocrine related presentations.	✓
Perform an appropriate systems-based physical examination of a patient for common respiratory, cardiac, gastrointestinal, neurological, genitourinary, vascular, abdominal, and endocrine related presentations.	✕
Summarise and interpret the findings obtained from the history and physical examination	✓ (omission of physical examination components)
List the differential diagnoses for common medical and surgical presentations.	✓
Outline the initial investigations for common medical and surgical presentations.	✓
Outline a management plan for common medical and surgical presentations.	✓
Demonstrate interviewing techniques that enhance motivation to change health risk behaviours	✕
Recognize the importance of multidisciplinary team management for patient care	✕

The CMS module learning outcomes and online program learning outcomes. ✓ = included, ✕ = not included. Motivational interviewing to change health risk behaviours was not included in the online program because of the short duration of time available to pivot online and the limitations of the available resources, as well as the time required to develop this complex skill. In addition, this particular learning outcome has never been assessed formally in the IC3 longcase. In a standard longcase examintion the student would not be able to carry out a motivational interviewing session on a health risk behaviour within the allocated time. Such a complex skill requires time, a hollistic approach and rapport with the patient. In reality, physicians engage in this type of consultation on multiple occasions and for longer durations. To do so in such a timely manner would be beyond the level expected of a third year medical student. This component of the CMS module was deferred until face-to-face teaching recommenced

**Fig. 1:**
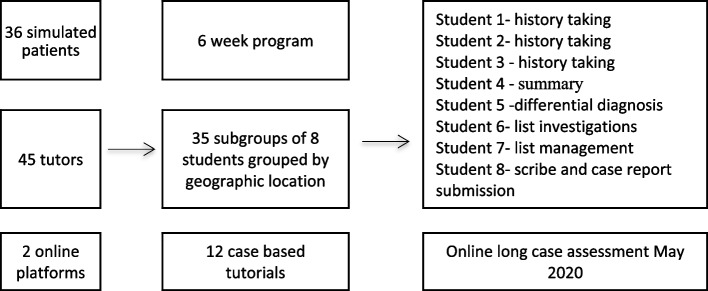
IC3 online teaching program design and resources used

The 275 students were divided into 35 subgroups of eight, each receiving 2 1-hour tutorials per week over a six-week period (cumulative run time of 420 hours). In order to account for time zone variation, students were grouped based on their geographical location at the time of the program. Each one-hour session involved eight students, a simulated patient and a clinical tutor with an additional ‘back-up’ clinical tutor and simulated patient on standby to offer support if any staff were unable to attend the session. Students were assigned clear roles in each tutorial such as history taking, summarising the history, formulating differentials, investigations and management (Fig. 1). Students from groups 1, 2 and 3 were mixed across tutorial groups so that there were varying degrees of clinical experience within groups. No students had completed the full clinical teaching program or the summative assessment of history taking skills (long case examination) (Table 1). The 10 weeks of clinical attachments are subdivided into different specialities and hospital groups, so at the time of COVID-19 related disruptions, each student was at a different stage of their own experiential learning path within their group. As such, we felt that all students would benefit from the full history-taking and communication skills teaching program to assist them in preparation for the long case examination, a high-stakes ‘must pass’ examination.

Rooted in both constructivist and social learning theories [14, 15], our online teaching program sought to create an interactive and learner centered approach [16], which would provide students with the skills to interview and diagnose patients, while applying knowledge gained previously, through constructive feedback from trained facilitators including peers. The structure of the online video link tutorial was modeled on the previously described Team Objective Structured Bedside Assessment (TOSBA), involving a small group of medical students undertaking a set of clearly defined clinical tasks and receiving immediate structured feedback on their performance [17]. Tasks were rotated between students after each session. Tutors observed tasks performed, provided structured feedback to each student and facilitated discussion among the group. The division of tasks in this manner created a constructively aligned learning opportunity to aid students’ preparation for their summative assessment, the year 3, semester 2 online long case examination (described below).

Following the tutorial, students were left in the Blackboard Collaborate meeting together to summarise the clinical case and complete a case report. This report was then submitted to the online student portal by the end of the day. In terms of educational resources, ‘model’ answers and video recordings of the tutorials were made available to each group on the following day. In addition, students were directed to use their year 3 semester 2 logbook. The logbook provided a list of ‘core patient presentations’ which students were expected to be competent in assessing by the end of the term (for example: a patient presenting with blood per rectum, haemoptysis, headache). This list included common medical and surgical problems, appropriate for the expected competency level of a third year medical student. They were also encouraged to use lecture notes from previous medicine and surgery modules in Year 2 and Year 3, which covered the presentation, investigations and management of common conditions.

### Educational methods and resources

Tutors involved in the teaching program included retired consultant tutors who volunteered to assist with medical education during the COVID-19 pandemic and non-consultant hospital doctor (NCHD) tutors employed by the university. In addition, newly qualified doctors from the RCSI graduating class of 2020 assisted as tutors prior to commencement of their internships (near-peer tutors). Retired consultant tutors or NCHD tutors taught over 50 % of tutorials. Newly qualified doctors assisted with the remaining tutorials. Tutors were rotated across groups so that students received equal exposure to experienced tutors and near-peer tutors. All tutors completed 1 hour of online platform and feedback training, in addition to receiving a tutor handbook and a Blackboard collaborate set up guide. In total, 36 simulated patients and 45 tutors took part. Online technical support was offered throughout the program.

Over the six-week teaching program an ‘organ systems based’ approach was used to facilitate integration of both medical and surgical specialties, and to provide a structured revision plan for the students. Faculty staff developed new clinical cases in the form of patient scripts and ‘model’ answers, which aligned directly with the existing curriculum (Examples provided in supplementary material). These were provided to each tutor and simulated patient prior to the online tutorials.

### Online examination structure

The online long case examination was similar in structure to the pre-COVID-19 long case examination structure, but with the omission of the physical examination component. Each student completed one medical or surgical long case examination virtually, the timing and content of which included:

- 12 minutes observed history taking
- 3 minutes of history presentation
- 5 minutes discussion of the case including differential diagnosis, investigations and management.
- 10 minutes changeover (allowing additional time for examiners to complete the mark sheet and allowing for any technical difficulties).

Of the 275 students that took part in the online program, 273 students sat the online long case assessment in May 2020. Students were examined over 2 days using the online platform Microsoft Teams (Microsoft Corporation, Redmond, WA, USA). As this was a summative assessment, only RCSI tutors and retired consultant tutors were used as examiners. All examiners and simulated patients were provided with online platform training, model answers and IT support. 12 virtual examination ‘stations’ were run simultaneously, with 12 examination circuits taking place on day one and 11 circuits on day two (cumulative run time 138 hours). An invigilator, a simulated patient and an examiner staffed each examination station. Examinations were recorded and discussed with senior examiners in a post examination debrief session. A second senior examiner reviewed borderline cases. In addition, as with face-to-face examinations, an external examiner was present on both examination days to ensure assessment integrity and standards were maintained. Students received an overall grade in accordance with the following categories:

- ≥70%, first class honors (1H)
- 65–69% second class honors grade 1 (2.1)
- 60–64% second class honors grade 2 (2.2)
- 50–59% pass (P)
- < 49% fail (F)

Students also received individual feedback concerning their performance of each of the 15 tasks or skills that were assessed within the online long case examination at a later date. Numerical scores were awarded to each student, however, for the purposes of this study results are presented as grade categories. In order to protect against identification of individual students, and to ensure compliance with data protection regulations, it is RCSI policy that numerical scores are transformed into categorical grades prior to analysis of examination results data, for projects that are to be submitted for publication.

### Validity

The only adaption made from the pre Covid-19 long case assessment was the removal of the ‘physical examination’ component. Each examiner was given a standardised mark sheet with predefined criteria relating to each section of the long case. The mark sheets provided an objective check list of competencies expected of the student. The expected competencies within each section of the long case mark sheet alligned with the original module learning outcomes (Table 2). The students were observed performing these skills under examination conditions. Students were graded as having performed each skill as “not done”, “not yet competent” or “done well” (see long case mark sheet in supplementary material).

### Reliability

Variability within the long case examination was reduced in a number of different ways: using standarsized timing, using standardised patients (actors with a predefined script) and standardised cases (approved by the academic department in Year 3 as appropriate for the expected level of competency). Inter-examiner variability was reduced through use of the standardised examination mark sheets and the grade guideline (see supplementary material). All students who were unsuccessful had their examination recording reviewed by a second examiner, again to reduce inter examiner variability. In addition, the external examiner who sat in on the examinations reported satisfaction with the conduct of the examination.

### Data collection and analysis

In order to evaluate the success of program, data was collected and analysed on student attendance, assessment results and student feedback on the program. Student demographic information, examination results, attendance and case-report submissions were obtained from departmental records and the University’s online portal system. The student survey was conducted by the RCSI Quality Enhancement Office, which performs annual student feedback analysis. The survey was anonymous, voluntary and sent to students via student email. It was not linked to any student assessments. Students were asked to complete a quantitative survey on the online teaching program and the University’s response to the COVID-19 pandemic. For the purposes of this study, only information relating to the online teaching program has been included. Regarding the online program, students were asked 11 quantitative questions and provided with a 5-point likert scale ranging from strongly disagree (SD) to strongly agree (SA). Microsoft Excel for Mac 2011 was used to analyse data and generate descriptive statistics regarding student attendance, examination results and student feedback (Microsoft Corporation, Redmond, WA, USA). Performance in examinations between groups and based on attendance profiles were compared using Pearson’s Chi-squared test. A *p*-value of 0.05 was used for statistical significance.

## Results

### Student attendance

**Fig. 2:**
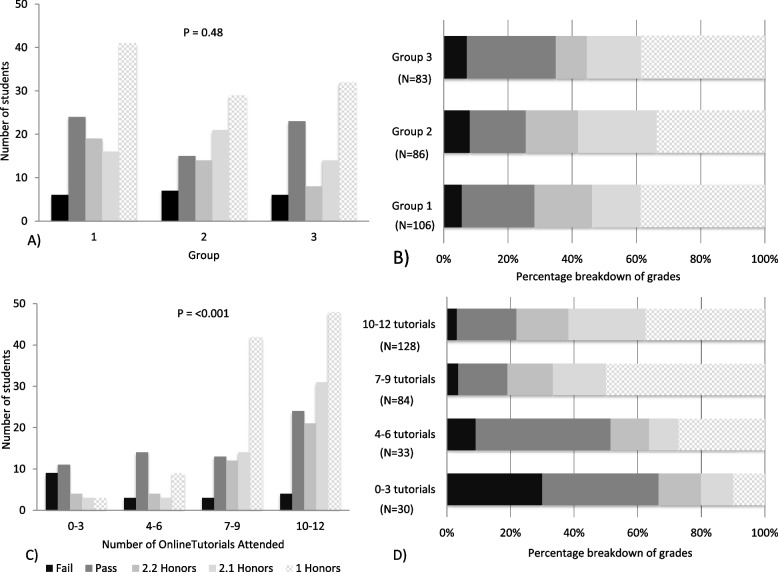
Grade distributions across groups and attendance profiles. **A)** The breakdown of grades in groups 1, 2 and 3 respectively. **B)** The percentage breakdown of grades in groups 1,2 and 3. **C)** The breakdown of grades based on attendance at online tutorials. **D**) The percentage breakdown of grades based on attendance at online tutorials

Mean attendance at tutorials for all students was 8 [3] of 12 tutorials (mean [standard deviation], 70% [27%]) (Table 3). Attendance was lower for males than for females (*p* <  0.001), and also lower for students located in the Middle East than for those located in other regions (*p* <  0.0001). Analysis by group demonstrated higher attendance rates for Groups 1 and 2 students compared with Group 3 students (*p* < 0.001) (Table 3 and Fig. 2).

**Table 3:** Summary of student attendance

	No. (%) students	% of Tutorials attended (***n*** = 12)	Between Group Difference ***P*** value	% of Case reports submitted (***n*** = 12)	Between Group Difference ***P*** value
**All students**	275 (100)	70 [27]		86 [14]	
**Sex:**					
**Male**	121 (44)	62 [31]	**0.0002**	86 [14]	**ns**
**Female**	154 (56)	76 [22]		87 [13]	
**Geographic location**					
**Europe**	58 (21)	75 [21]	**< 0.0001**	86 [14]	**ns**
**N. America**	59 (21)	83 [15]		83 [12]	
**Far East**	41 (15)	82 [24]		87 [12]	
**Middle East**	116 (42)	56 [30]		87 [13]	
**Rotation**					
**Group 1**	106 (39)	75 [24]	**0.0002**	87 [13]	**ns**
**Group 2**	86 (31)	73 [24]		86 [15]	
**Group 3**	83 (30)	60 [31]		86 [14]	

Data provided as number (%) or mean % [standard deviation %]

Case report submission rates were calculated for subgroups, not individual students

The average case report submission rate was 10 [2] of 12 (86% [14%]) and there was no significant difference in submission rates across subgroups. Case report submission rates were calculated for the 35 subgroups rather than for individual students. As this was collaborative group work submitted by one individual student in each subgroup, it was not considered a representative indictor of individual student engagement.

### Assessment results

A total of 273 students completed the Year 3 Semester 2 online long case examination in May 2020. Total grade breakdown was as follows: 1st class honors (1H) *n* = 102 (37%), 2nd class honors grade 1 (2.1), *n* = 51 (19%), 2nd class honors grade 2 (2.2), *n* = 41 (15%), pass (P), *n* = 62 (23%), fail (F), *n* = 19 (7%). Interestingly, grade distribution was similar across groups 1,2 and 3, despite the differing levels of clinical experience prior to the online program commencing (Fig. 2, panels A and B) (*p* = 0.48).

By contrast, increased attendance rates at tutorials were associated with higher numbers of honors grades (Fig. 2, panels C and D) (*p* < 0.001). 78% of students who attended 10–12 tutorials achieved an honors grade, whereas only 34% of those who attended 0–3 tutorials were awarded honors. Similarly, there were higher rates of fail grades awarded to students who only attended 0–3 tutorials (30%) than those who attended 10–12 tutorials (3%).

### Student feedback

The response rate to the student survey was 31%. Overall, the students demonstrated considerable satisfaction with all aspects of the online program. The majority of respondents agreed or strongly agreed that they had the opportunity to ask questions (93%), that the tutorials were interactive (86%) and that the tutorials increased their understanding of the course material (81%). Most students surveyed were satisfied with the staff facilitation of the program (88%). Many agreed or strongly agreed that staff were familiar with the technology (82%) and provided clear instructions (86%). Most students reported good connectivity and sound throughout the online teaching sessions, and many were satisfied with the usability of the program (80, 77, 89% respectively). In terms of the overall online delivery approach, the majority of respondents felt that their queries were addressed (72%), and that contacts or sources of further information were well signposted (88%) (Table 4).

**Table 4:** Quantitative feedback regarding the online teaching program, platform technology and faculty support

Student Survey Questions	Percentage agreement (A or SA)	Mean (standard deviation)
A. I had the opportunity to ask questions during online classes	93%	4.36 (1.1)
B. I interacted with other students during online classes	86%	4.16 (0.98)
C. I was satisfied with the facilitation of the online classes	88%	4.11 (1.02)
D. Online lectures/tutorials increased my understanding of the materials in the module	81%	4.02 (0.91)
E. The staff were familiar with the platforms used to deliver the live classes	82%	4.1 (0.92)
F. I received clear communication regarding what I was expected to do at all times for online classes	86%	4.11 (0.99)
G. I was generally able to maintain a good connection with the platform through the live sessions	80%	3.96 (0.9)
H. The sound quality was good throughout the online sessions	77%	3.92 (0.84)
I. The technology used for online classes was easy to use	89%	4.26 (1.05)
J. I received responses to any queries I made	72%	3.91 (0.81)
K. I was aware of channels of communication I could use to have any questions I had answered	88%	4.13 (1.11)

Student survey results including percentage general agreement with statements (strongly agree or agree) and mean agreement (standard deviation) where strongly disagree (SD) = 1, disagree (D) = 2, neither agree nor disagree (N) = 3, agree (A) = 4 and strongly agree (SA) = 5

## Discussion

Many articles have described experiences of pivoting case based teaching online, the use of flipped classrooms, the use of personal protective equipment (PPE), didactic online lectures and simulation technology in response to the COVID-19 pandemic [8, 18–20]. In addition, authors have described virtual rotations delivered via online platforms [21], the development of simulated scenarios [22] and standardized patient encounters [23]. These innovations were developed to aid the development of important history and clinical reasoning skills for learners. While much theoretical literature exists for delivery and assessment in procedure based simulation [24], little evidence exists for this in the teaching of core clinical reasoning skills in undergraduate medical education [25]. Previous work has demonstrated that clinical skills can be acquired virtually and there is no difference in acquisition between students who have had online or more traditional teaching [26]. Here we have described our single center experience of an online case based teaching program developed rapidly in response to the COVID-19 pandemic. A number of educational methods were used to optimise learning during the online program.

### Small group teaching

As mentioned previously the structure of the online video link tutorial was modeled on the previously described Team Objective Structured Bedside Assessment (TOSBA), involving a small group of medical students undertaking a set of clearly defined clinical tasks and receiving immediate structured feedback on their performance [17]. The division of tasks in this manner created a constructively aligned learning opportunity (Fig. 1, Table 2). The use of structured feedback helped to focus attention on tasks done well and tasks requiring improvement. Used in this way, constructive feedback can both reinforce and adapt behaviours, prompting reflection and improving student performance [27]. Other groups that have implemented small group teaching programmes during the COVID-19 pandemic have demonstrated no impact on learning outcomes of students when engaging in online learning compared to face-to-face learning [28, 29].

### Synchronous and asynchronous learning

In addition to the live tutorials, multiple learning tools were available to students including case report submissions, model answers and recordings of each tutorial. This use of both synchronous and asynchronous learning tools aimed to support different learning styles, increase learning flexibility and provide an opportunity for consolidation and reflection outside of the live teaching sessions. Worked examples have been shown to provide a ‘diagnostic schema’ for students, which augments the acquisition of diagnostic skills [30]. The model answers provided a worked example of how to summarise the pertinent positive and negative points in a particular case, for example: describing a history of shortness of breath associated with a productive cough (relevant positive) but also mentioning the absence of haemoptysis or weight loss (relevant negatives). The model answers also provided the clinical reasoning behind the differential diagnoses (including how and why to rule out certain differentials) and the reasoning behind investigations and management (Supplementary material). Other educators have published examples of synchronous and asynchronous learning during the pandemic. A similar approach to the one outlined in this paper was taken by one group who provided pre reading and pre-test material to students. The authors found that this model empowered students’ engagement and interactive learning [31]. Another study that looked at the impact of such learning techniques found that synchronous learning allowed for more social integration and feedback with students and fostered greater psychological wellbeing [32]. While this was not studied in our current study it will be taken into consideration in future curriculum planning.

### Near-peer based teaching

While the effect of using newly graduated doctors as tutors was not measured in this study, the positive effects of near-peer based teaching are well established [33–35]. Near-peer teachers have a similar knowledge base to the learners, and thus can identify more closely with the struggles of students, creating a safe environment to make mistakes and to ask questions [36]. Given the large scale of our educational development, the use of newly qualified graduates as peer tutors helped us to implement this program rapidly, reducing teaching burden when faced with limited resources. In addition, given the high level of interaction and task distribution within the online tutorials, it is likely that some reciprocal peer teaching and learning took place also. Unfortunately this was not explored directly in the student survey. All students observed each other performing allocated tasks within the case, following which there was a discussion among the group regarding outstanding questions, points missed, differential diagnoses and management. Previous studies have reported student satisfaction with reciprocal-peer teaching models, demonstrating satisfactory knowledge acquisition through teaching and learning [37, 38].

### Student attendance and assessment results

Despite the lack of a mandatory attendance policy, mean attendance on the online program was high at 70%. There was a wide variety of clinical experience across the three groups of students prior to the commencement of this online program (Table 1). Despite this, grade distribution was similar across all groups of students (Fig. 2). Interestingly, student attendance was lower in Group 3 compared with Groups 1 and 2. Group 3 students had gained the most clinical experience, having completed seven out of 10 weeks of clinical attachment and the summative clinical skills exam (Objective Structured Clinical Examination- OSCE). Prior experience gained may account for the similar grades despite the reduced attendance of Group 3.

### Student feedback

There was significant satisfaction with the format of the online program reported by students. Particular areas of note included how interactive the sessions were and how the program augmented their understanding of the module material. Given that poor internet connectivity and lack of faculty training are some of the barriers to online education commonly reported [39], it is positive that survey participants reported good connectivity and sound quality on the platforms used. They also reported satisfaction with staff facilitation of the online platforms. It is important to note that the survey uptake rate, at 31%, was relatively low. Thus, the above positive feedback may be subject to participation bias.

### Future implications

The RCSI has a number of geographically dispersed clinical teaching sites, including large and small teaching hospitals. The availability of dedicated university tutors varies across sites and currently a mobile tutor travels to smaller hospitals to provide onsite clinical teaching for year 3 students. We believe that this online program could be used in the future to facilitate distance teaching and learning across a range of geographically separated clinical teaching sites, which would prove more economically and environmentally feasible. In addition, new or resurging pandemics pose an ongoing threat to the future of medical education. Over the last 20 years we have witnessed the emergence of serious infectious outbreaks such as SARS, MERS, Ebola and H1NI [40]. The potential for periodic disruption to medical education in the future is likely. Therefore, fluency in telemedicine and the use of remote teaching such as this program should be embedded within medical school curricula.

### Strengths

The strength of this online program lies in the scale of the program and the speed at which it was implemented. Within 3 weeks the faculty team developed additional clinical case content, recruited additional teaching staff and designed a schedule to facilitate students located across different continents. Following completion of the program, 93% of students successfully passed the year 3 semester 2 online long case examination, facilitating onward progression through the degree program without delay. Furthermore, at a time of great uncertainty and isolation, the online teaching program provided an opportunity for structured revision remotely, social interaction and connectedness among students. As part of the student survey 86% of respondents confirmed that they had interacted with other students during the online tutorials (Table 4).

### Limitations

While the results of student feedback suggested significant satisfaction with the program, it would be beneficial to assess students’ experience of the online program relative to in-person teaching, the effect of peer tutor teaching, psychological wellbeing while using such platforms and to gain formal feedback from tutors. Ultimately, the use of simulated patients in isolation to develop clinical skills does not replace real patient encounters. In addition, the remote nature of this program prevented teaching of clinical examination skills. In our university, students in Group 1 and Group 2 went on to take part in face-to-face clinical teaching and clinical attachments in order to complete the remaining program requirements, once local restrictions were lifted in September 2020.

### Conclusions

The COVID-19 pandemic created significant disruption to the delivery of medical education globally. The seismic effort to implement rapid change as a result has created an extraordinary learning opportunity for the educational community. We have shared our experience of the development, implementation and delivery of an online teaching program. We have described in detail how the use of online video technologies, simulated patients and tutors with varied experience can be used to develop clinical reasoning skills remotely. Our aim was to continue clinical teaching and learning opportunities remotely during the COVID-19 pandemic, and as a result 93% of students successfully passed the online long case examination, facilitating onward progression through the undergraduate medical program. This online program could be replicated and used again to overcome some of the barriers to medical education stemming from the pandemic. In addition, this program could be used to facilitate distance teaching and learning across a range of distant clinical teaching sites in the future.

## Supplementary Information

**Additional file 1:** **Supplementary Table 1 (S1).** Sample case report.

## Data Availability

The datasets generated and analysed during this study are not publicly available due to privacy concerns but are available from the corresponding author on reasonable request. A sample case, its model answer and case report are provided in the supplementary material, as are the long case examination mark-sheet and grade guideline.

## Abbreviations

RCSI: Royal College of Surgeons Ireland
SSP: Student selected project
OSCE: Objective structured clinical examination
MSK MCQ: Musculoskeletal multiple choice question
CMS: Clinical medicine and surgery
PPE: Personal protective equipment
SARS: Severe acute respiratory syndrome
MERS: Middle east respiratory syndrome
H1N1: Influenza A H1N1
1H: First class honors
2.1: Second class honors grade one
2.2: Second class honors grade two
P: Pass
F: Fail
SD: Standard deviation
