# Supplementary material for: An online case-based teaching and assessment program on clinical history-taking skills and reasoning using simulated patients in response to the COVID-19 pandemic
Source: BMC Med Educ. 2023 Jan 4;23:4. doi: 10.1186/s12909-022-03950-2 (PMC9811710; doi:10.1186/s12909-022-03950-2)
Supplement: Supplementary file 1 — Additional file 1: Supplementary Table 1 (S1). Sample case report. [file 12909_2022_3950_MOESM1_ESM.docx]

**
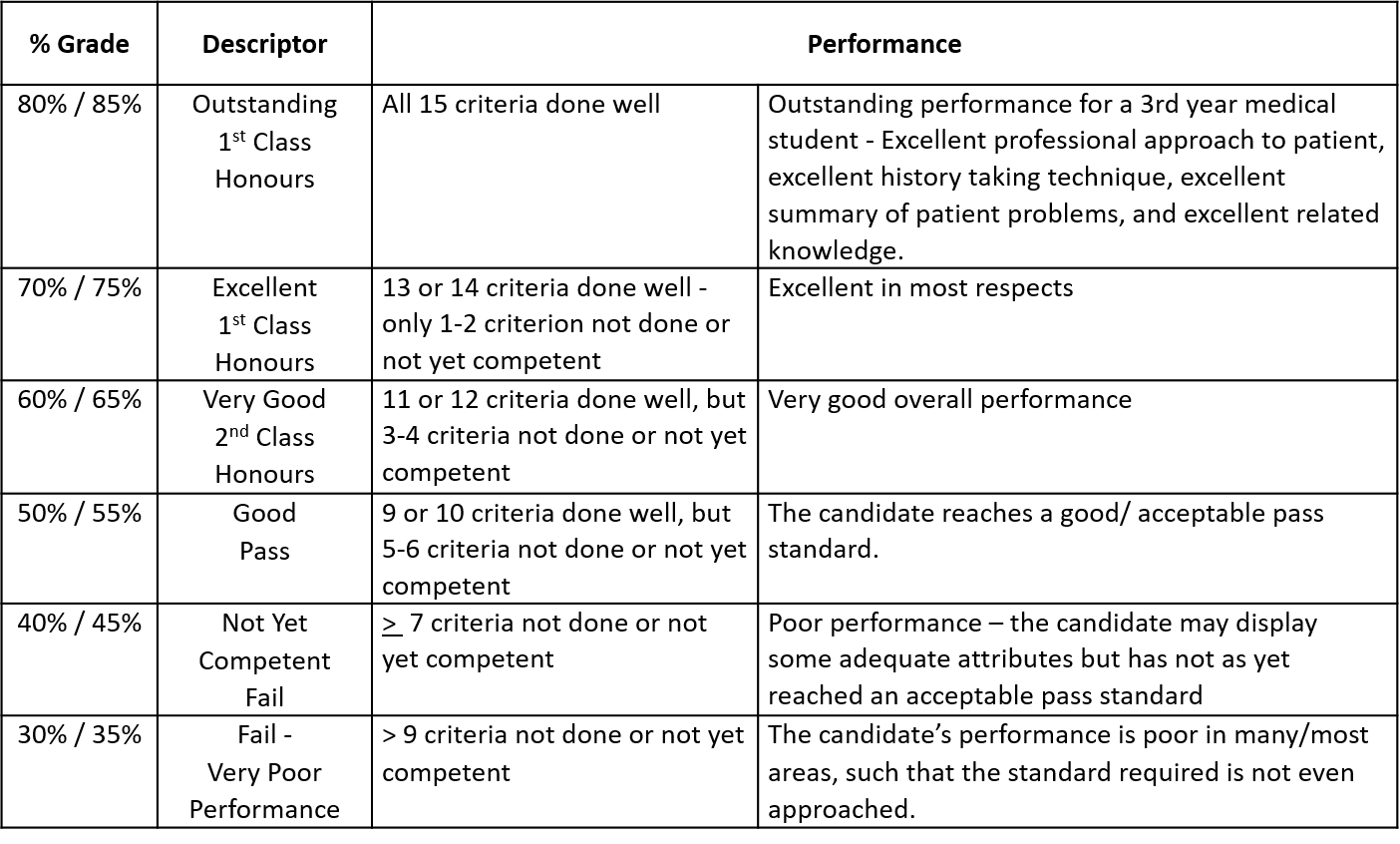
IC3 2020 Long Case Grade Guideline**

**Respiratory Case 2 (Pneumonia)**

**History**

**Starting Student Number = 5**

**Student to be told by Tutor/Facilitator:**

You are working as an emergency department doctor. This patient has been referred by their general practitioner with a cough. Please begin with history taking.

**__________________________________________________**

**Instructions for Actor:**

- This is an assessment of information gathering as opposed to dealing with an anxious patient.
- The student will ask questions related to the chest, breathing, cough and infection.

**Background story for Actor…**

**Charles/Charlotte A. Purcell**

- 50 year old taxi driver from Santry (nearby for Perdana/Bahrain)
- You went to the GP this morning because you “couldn’t shake the cough”
- If asked why GP referred you onwards…
  - The GP took one look at you and sent you in
  - “didn’t like how I looked”
  - “said I was very sick”

**Presenting Complaint:**

- Shortness of breath
- Coughing up dark green phlegm
- Feverish

**History of Presenting Complaint**:

Ideally you will wait to be asked about cough, fever, sputum, chest pain and shortness of breath before elaborating but if student stuck keep going back to your worry about “the cough”

If asks a specific question not covered below, feel free to ask the student to repeat the question or rephrase or “what do you mean” or just say no/deny the symptom.

- Onset of problems:
  - Cough started about two weeks ago
  - But really deteriorated about three days ago
- Shortness of breath:
  - Your breathing has been getting worse for the last week
  - Sometimes you have a hard time catching your breath after coughing
  - Get short of breath going up a flight of stairs now, which usually causes you no issues
  - Get short of breath if talking for an extended period of time.
- Cough:
  - You are coughing up large quantities of green phlegm – a tablespoon every half an hour
  - Your phlegm is thick and green in the last few days
  - Previously it was a bit yellow for a week or so
  - No particular trigger
- Fever:
  - You felt hot and sweaty a few times in the last week.
  - You checked your temperature and noted that it has been over 38 degrees for the last 2 days
- Overall:
  - Lots of muscle and joint pains
  - You tried to sleep it off but it is getting worse
  - You called to your GP, who examined you and then sent you to hospital

***If asked specifically:***

- It makes no difference to your breathing if you are sitting up or lying down.
  - You do not need extra pillows or wake up in the middle of the night to catch your breath
- Your ankles aren’t swollen/puffy
- **You have developed a wheeze**
- You had a chest infection 5 months ago but it wasn’t as bad as this. Your GP prescribed you a course of oral antibiotics
- You have never been admitted to hospital before.
- You have not noticed any weight loss.
- You have never coughed up any blood.
- It’s worse in the evening.
- You don’t have any sinus problems and have not been hoarse recently.
- Your appetite has been fine.
- You have had no vomiting or diarrhoea.
- No asbestoses exposure
- No pets, budgies, etc
- No recent travel abroad
- Everyone else you know is currently well
- Never been diagnosed with “COPD”

**Past Medical and Surgical History:**

- Medical History
  - You have diabetes mellitus for the last 3 years
  - You have high blood pressure for 5 years
  - You get the flu vaccine every year
  - You have never seen a lung specialist.
  - You have high cholesterol.
- Surgical history:
  - Nil

**Medications/Allergies:**

- You have a severe penicillin allergy… throat closes, facial rash, swollen lips, ended up in a “intensive unit”… happened 5 years ago…
- You take…. Glucophage/metformin for the diabetes
- You take… Tritace/Ramipril for your high blood pressure
- You take… Atorvastatin for your cholesterol
- If forget names of medications: Just say “a tablet for my cholesterol” etc.

**Social History:**

- You don’t smoke now.
  - You used to smoke about twenty a day since you were 20 years old but you stopped 5 years ago
- You drink socially, but not regularly. At most once per week, 4-5 glasses of wine
- You live with your husband/wife, who is well and working in the bank
- Live in Bungalow

**Family History:**

- Your father died of old age, aged 95.
- Your mother died of a heart attack aged 80.
- You have two siblings who are both well and living nearby. Your brother was recently diagnosed with diabetes

**Review of Systems**

- No additional symptoms or complaints

**Respiratory Case 2 (Pneumonia)**

**Model Answer**

**Sample summary statement:**

“In summary, Mr/Mrs. Purcell is a 50-year-old taxi driver from Dublin, referred by his/her GP with a 2 week history of worsening productive cough, associated more recently with fever, shortness of breath and muscle aches, on a background of a twenty five pack year smoking history.

**Based on this, my differentials include:**

1. Pneumonia

- Acute onset productive cough (green sputum) and fevers. In addition, this patient has not had the pneumococcal vaccine.

2. Atypical pneumonia (eg. Legionella)

- A less common pathogen causing community acquired pneumonia, but should be considered.

3. Influenza or COVID-19

- Fever and myalgia are both features of influenza. Other expected symptoms would include sore throat, malaise, headache, and dry cough. While he/she has had the influenza vaccine, efficacy can vary depending on variations in seasonal viral strains so it should still be considered in the differentials.
- COVID-19 typically presents with a cough, sore throat, fever, breathlessness, loss of taste & smell, and also myalgias and fatigue

4. Infective exacerbation of COPD

- Significant smoking history, productive cough and wheeze (may have undiagnosed COPD).

**Rationale for excluding other differentials:**

Other infective causes (fungi, mycobacterium, abscess)- less likely due to absence of weight loss, night sweats, recent travel (TB, abscess) and significant immunosuppression (fungi). Note- diabetes mellitus is considered relative immunosuppression, not significant.

Bronchiectasis- The absence of chronic productive cough or recurrent chest infections makes this less likely. In addition, the absence of risk factors such as previous infection (eg. TB, Measles, pertussis) or underlying conditions (eg. cystic fibrosis, alpha1anti-trypsin), make this less likely.

Malignancy- While there is a significant smoking history, the acute nature of these symptoms and absence of red flags such as haemoptysis, weight loss and hoarseness, make this less likely.

**Investigations:**

It is important when presenting your investigations to consider beginning with relevant non-invasive bedside tests and then moving to more invasive tests. For example – we do not begin with a CT thorax before taking a sputum sample. **You should explain and justify why you are considering doing each test.**

Investigations in the emergency department:

Following completion of a full history and respiratory examination I would like to…

- Check the patient’s vital signs and oxygen saturations
- Obtain a sputum sample (microscopy culture + sensitivity + cytology)
- Urine antigens: legionella, pneumococcal
- Viral swab (influenza + covid-19)
- In the context of shortness of breath- perform an ECG to assess for structural/Ischaemic changes/ arrhythmias (cardiac disease)/right ventricular strain pattern (pulmonary disease)
- Perform bloods
  - Full blood count (FBC): to look at white cell count for evidence of infection
  - Urea and electrolytes: To assess baseline renal function and evidence of an acute kidney injury in the setting of infection
  - Liver function tests: to assess baseline function prior to commencement of antimicrobials, can be deranged in legionella and mycoplasma infection
  - C reactive protein (CRP): as a marker of infection and inflammation
  - Arterial blood gas (ABG): if oxygen saturations are low, to assess for respiratory failure
  - Blood cultures if features of sepsis are present on admission, to identify pathogens
- Chest X-ray: to assess for evidence of the cause: consolidation, air bronchogram, effusion (pneumonia), bullae, hyperinflation (COPD).

**Management:**

- CURB-65 score determines need for admission: a score of > 2 requires admission to hospital
  - **C**onfusion 1
  - **U**rea >7mmnol/L 1
  - **R**espiratory rate >30 breaths per min 1
  - **B**lood pressure (systolic <90mmHg or diastolic <60mmHg) 1
  - **65** – age >65 1
- Antibiotics (amoxicillin + clarithromycin), oxygen and fluids as required, chest physiotherapy, DVT prophylaxis
- Repeat chest x-ray after 6 weeks to ensure resolution
- Preventive: pneumococcal vaccine and influenza vaccine

| **Case: Respiratory** | | |
| --- | --- | --- |
| **Tutorial Group** | **Students** | **Attendance (present/absent)** |
|  | **1.** |  |
|  | **2.** |  |
|  | **3.** |  |
|  | **4.** |  |
|  | **5.** |  |
|  | **6.** |  |
|  | **7.** |  |
|  | **8.** |  |
| **Case Summary** | | |
| **Differential Diagnoses** | | |

Supplementary table 1 (S1). Sample case report
